# Vertically aligned P(VDF-TrFE) core-shell structures on flexible pillar arrays

**DOI:** 10.1038/srep10728

**Published:** 2015-06-04

**Authors:** Yoon-Young Choi, Tae Gwang Yun, Nadeem Qaiser, Haemin Paik, Hee Seok Roh, Jongin Hong, Seungbum Hong, Seung Min Han, Kwangsoo No

**Affiliations:** 1 Department of Materials Science and Engineering, Korea Advanced Institute of Science and Technology (KAIST), Daejeon 305-701, Korea; 2 Materials Science Division, Argonne National Laboratory, Lemont, IL 60439, USA; 3 Graduate School of Energy Environment Water Sustainability, Korea Advanced Institute of Science and Technology (KAIST), Daejeon 305-701, Korea; 4 Nuclear Engineering Division, Argonne National Laboratory, Lemont, IL 60439, USA; 5 Department of Chemistry, Chung-Ang University, Seoul, 156-756, Korea

## Abstract

PVDF and P(VDF-TrFE) nano- and micro- structures have been widely used due to their potential applications in several fields, including sensors, actuators, vital sign transducers, and energy harvesters. In this study, we developed vertically aligned P(VDF-TrFE) core-shell structures using high modulus polyurethane acrylate (PUA) pillars as the support structure to maintain the structural integrity. In addition, we were able to improve the piezoelectric effect by 1.85 times from 40 ± 2 to 74 ± 2 pm/V when compared to the thin film counterpart, which contributes to the more efficient current generation under a given stress, by making an effective use of the P(VDF-TrFE) thin top layer as well as the side walls. We attribute the enhancement of piezoelectric effects to the contributions from the shell component and the strain confinement effect, which was supported by our modeling results. We envision that these organic-based P(VDF-TrFE) core-shell structures will be used widely as 3D sensors and power generators because they are optimized for current generations by utilizing all surface areas, including the side walls of core-shell structures.

Organic ferroelectric nano- and micro-structures have attracted significant interest due to the widespread use of the portable^1^, implantable^2^, and flexible electronic devices^3^. Among the numerous ferroelectric polymers, poly-(vinylidene fluoride) (PVDF) and its copolymer poly-(vinylidene fluoride-co-trifluoroethylene), (P(VDF-TrFE)) are strong candidates for these types of practical applications due to their relatively large remnant polarization values^4^, high piezoelectric voltage constants^5^, low dielectric constants^6^, and high chemical stability^2^.

Recently, many research groups have focused on enhancement of piezoelectric energy conversion efficiency from mechanical to electrical energy using these materials. Wang and Cha *et al.*^7^ introduced porous PVDF nanogenerators that are fabricated via a template-assisted method, where superior power generation characteristics under the same sonic wave compared to the bulk PVDF film nanogenerators were reported. The enhanced piezoelectric potential was attributed to the geometrical strain confinement effect from porous PVDF nanostructures. In another study, Chang *et al.*^8^ synthesized PVDF nanofibers on flexible substrates with *in situ* electromechanical poling using the near-field electrospinning (NFES) technique, and, interestingly, individual PVDF nanofibers were reported to have higher energy conversion efficiency than the PVDF film. However, although it is known that the piezoelectric effects can be enhanced by fabricating the material into nano- and micro-structures, relatively few studies have been undertaken for the fabrication and characterization of vertically aligned nano- and micro-structural piezoelectric polymer energy scavenging materials, as their low stiffness makes such nano- and micro- scale pillar array fabrication extremely difficult.

Synthesis of nano- and micro- scale piezoelectric polymer structures were previously attempted using a variety of methods such as electrospinning^8^,^9^, thermal imprinting^10^, and a template-assisted method^5^,^7^,^11^. The electrospinning process produces a high degree of crystallinity and aligns the chain orientation during the electrospinning stage; however, the individual fibers are horizontally deposited on the substrate that makes effective z-axis straining difficult. The thermal imprinting process is also a feasible fabrication method for vertically aligned and uniformly patterned structures; however, the polarization of the coated film is abruptly and irreversibly reduced during the melting and re-crystallization processes^12^. Lastly, the template-assisted method using anodic aluminum oxide (AAO) or a Si mold can easily fabricate one-dimensional structures, but this method is not practical for mass production because of the need to sacrifice the template.

In this study, a simple and cost effective method of fabricating vertically aligned P(VDF-TrFE) core-shell micropillars, in which Pt-coated PUA pillar structures are used as cores and P(VDF-TrFE) nanometer scale-thick layers are used as shells, is introduced. The P(VDF-TrFE) is compliant with Young’s modulus of 1.5 GPa^13^ that makes synthesis of nanoscale structure difficult^5^. However, P(VDF-TrFE) with nanoscale thickness can be fabricated by depositing the P(VDF-TrFE) on top of the PUA micropillar, which has Young’s modulus of 1.6 GPa^14^, that is used as a mechanical support. The converse piezoelectric coefficient of the P(VDF-TrFE) core-shell structures were then evaluated from the displacement of the individual micropillar as a function of applied bias voltage under a constant compressive stress using a nano-indenter (nano-ECR, Hysitron) equipped with an *in-situ* electrical biasing circuit. Therefore, the piezoelectric energy generation efficiencies were inferred and analyzed from the converse piezoelectric coefficient of each micropillar to evaluate the effectiveness of the proposed P(VDF-TrFE) core-shell micropillar structure.

## Results and Discussion

**Figure 1:**
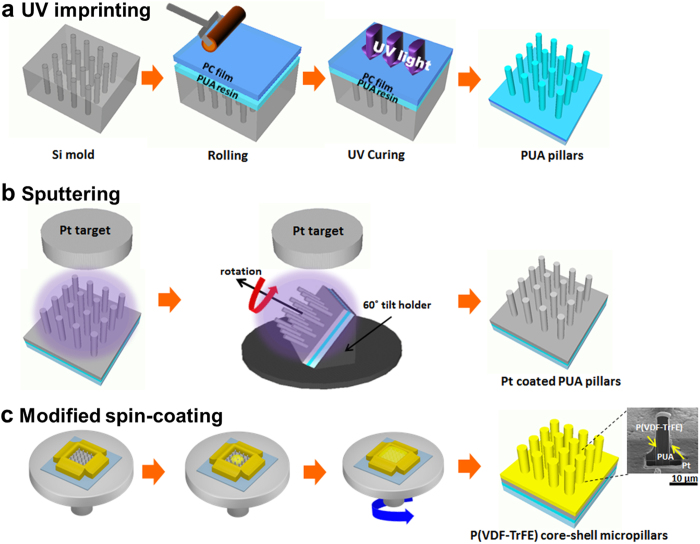
Schematic illustration of the fabrication of the P(VDF-TrFE) core-shell micropillars. (**a**) The PUA micropillar arrays were fabricated via a UV imprinting process and (**b**) the Pt bottom electrode was deposited on the PUA pillars via a sputtering process. (**c**) The P(VDF-TrFE) solution was spin coated onto the Pt coated PUA pillars.

The vertically aligned P(VDF-TrFE) core-shell micropillars were fabricated via a three-step process, which includes UV imprinting, sputtering and modified spin coating process, as illustrated schematically in Fig. 1a–c.

The UV-cured polyurethane acrylate (PUA) elastomer was coated onto a Si master, which was prepared via conventional lithography and a reactive ion etching (RIE) process. Then, the PUA elastomer was covered with a back-supporting polycarbonate (PC) film, which was subsequently exposed to a UV light. The UV-cured PUA micropillar arrays with a 5 μm diameter, a 20 μm height, and a 30 μm pitch were precisely replicated from the complementary micro-patterned Si master mold as shown in Fig. 1a. In Fig. 1b, the platinum layer was deposited on the surface of the PUA pillar arrays as the bottom electrode using the sputtering deposition process. In Fig. 1c, the piezoelectric P(VDF-TrFE) layer was then deposited onto the Pt-coated PUA pillar arrays using the modified spin-coating process with a spin speed of 600 rpm for 10 s. Subsequently, the sample was annealed at 125 °C for 2 h (see more detailed information in the methods section).

**Figure 2:**
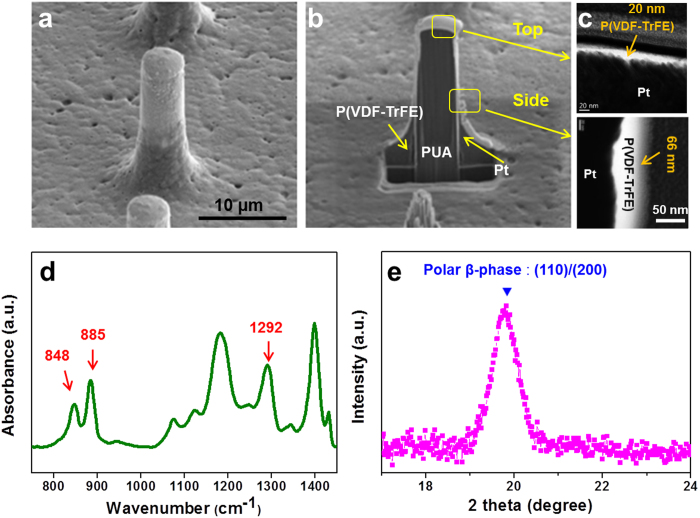
Thickness analysis and ferroelectric properties of P(VDF-TrFE) core-shell micropillars. SEM images of P(VDF-TrFE) core-shell micropillars (**a**) before and (**b**) after the FIB milling. (**c**) Cross-sectional TEM images that confirm the presence of a nanoscale thin layer of P(VDF-TrFE) on both top part and side walls of the micropillar. (**d**) FT-IR spectra, and (**e**) XRD analysis of the P(VDF-TrFE) core-shell micropillars.

The scanning electron microscopy (SEM) images and energy dispersive spectroscopy (EDS) spectrums of the Pt-coated PUA pillars and P(VDF-TrFE) core-shell arrays are provided in Fig. S1. Fig. 2a,b present the cross-sectional SEM images of the P(VDF-TrFE) core-shell micropillar before and after the focused ion beam (FIB) milling, respectively. Fig. 2b clearly indicates that the Pt layer was continuously deposited on the PUA pillars. The P(VDF-TrFE) layers were coated onto the Pt/PUA pillars and the meniscus lines were formed on the edge of the core-shell structure. The thicknesses of the top and the side wall of the continuous thin P(VDF-TrFE) film on the core-shell micropillar are 20 nm and 66 nm, respectively. Fig. 2c shows the cross-section transmission electron microscopy (TEM) images of the P(VDF-TrFE) core-shell micropillar at the top and the side wall of the pillar.

Fourier transform infrared (FTIR) analysis was performed on the P(VDF-TrFE) core-shell structures to confirm the all-trans configuration of the ferroelectric β-phase as shown in Fig. 2d. The absorption bands were confirmed at 1292 cm^–1^, 848 cm^–1^ (A1, ***μ***|| ***b***), and 885 cm^–1^ (B2, ***μ***|| ***a***), which are related to the ferroelectric polar phase possessing all-trans sequences. The absorption bands at 1195 cm^−1^ and 1402 cm^−1^ are related to the anti-symmetric stretching vibrations of CF_2_ and to the orientation of the carbon main chains, respectively^15^,^16^,^17^. An X-ray diffraction (XRD) analysis of the P(VDF-TrFE) core-shell arrays confirmed that the (110) and (200) reflections from the crystalline β-phase of P(VDF-TrFE) were present (Fig. 2e)^18^.

**Figure 3:**
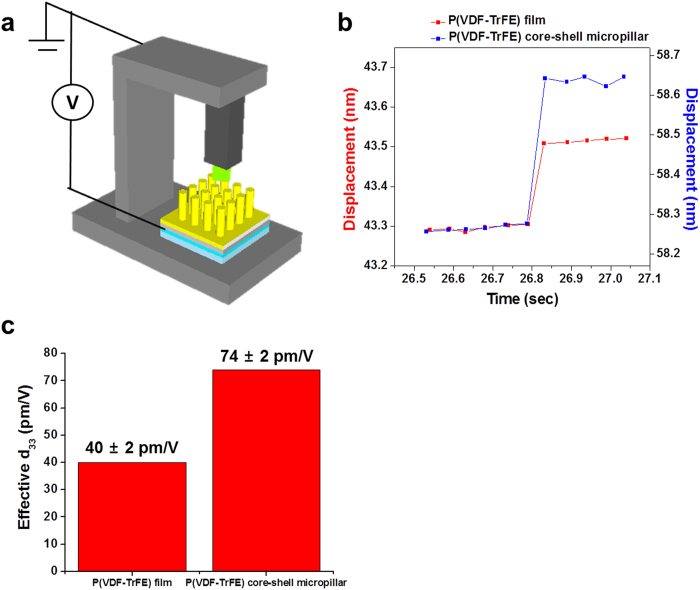
Characterization of converse piezoelectric constant d_33_ of P(VDF-TrFE) thin film and core-shell micropillar using a nanoindenter. (**a**) Schematic illustration of the setup for the characterization of the converse piezoelectric effects using a nanoindenter. (**b**) The displacement changes of the P(VDF-TrFE) film (red squares) and the core-shell micropillar (blue squares) induced by an electric bias voltage of 5 V. (**c**) The effective converse piezoelectric constant d_33_ of the P(VDF-TrFE) film and the core-shell micropillar.

For the characterization of the converse piezoelectric properties of individual P(VDF-TrFE) core-shell micropillars, we used the micropillar compression method with *in-situ* electrical biasing (NanoECR, Hysitron) as shown in Fig. 3a. A pulsar tip with a diameter of 8.3 μm was used to perform the compression tests on a P(VDF-TrFE) core-shell micropillar with a diameter of 5.4 μm. To ensure that the tip made a full contact with the micropillar, the contact area between the pulsar tip and the top of the pillar was calculated from the stiffness extracted from the unloading slope in the force-displacement curve, as explained in the Supporting Fig. S2. In addition, the modulus (2.5 GPa) calculated from the unloading slope was in close agreement with the theoretical modulus (2.1 GPa) assuming iso-stress condition (Eq. S5), thereby confirming the validity of the pillar indentation results.

Firstly, we attempted to measure the direct piezoelectric effect via measurement of current generated during the indentation^19^, however we found that NanoECR instrument has an internal leakage current, which makes the measurement of the direct piezoelectric effect inaccurate (see Fig. S3). Therefore, we measured the converse piezoelectric effect using the voltage-displacement analysis^20^. As such, we measured, with a position accuracy of 0.01 nm, the displacement of the nano-indentation tip in contact with either a 1 μm thick P(VDF-TrFE) film or a core-shell micropillar while applying the bias voltage between the tip and the bottom electrode.

During the measurement of the converse piezoelectric effect, we applied the trapezoid load function with loading-hold-unloading segments where the maximum load was maintained at the force of 100 μN as shown in Fig. S4b. In order to measure the surface displacement induced by the applied voltage, we applied 5 V at 26.8 seconds for 10 seconds and measured the change in the displacement during the hold segment with a constant load of 100 μN. The reason we applied 100 μN without a voltage bias for 26.8 seconds before we measured the converse piezoelectric induced displacement was to reach a steady state in the creep deformation of the polymers (Fig. S4).

The displacement changes of the P(VDF-TrFE) film and the core-shell micropillar induced by 5 V at 100 μN were 0.2 nm and 0.37 nm, respectively, as shown in Fig. 3b. We calculated the effective converse piezoelectric constant, d_33_^eff^, using Eq. (1).
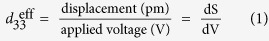
where dS is the change in displacement, and dV is the change in the applied voltage bias. The average values of effective d_33_’s of P(VDF-TrFE) film and core-shell micropillar, which were measured 10 times, were 40 ± 2 pm/V and 74 ± 2 pm/V, respectively (see Fig. 3c).

To further confirm the enhancement of the effective piezoelectric constant d_33_ in core-shell micropillars over a wide range of the loading force, we measured the effective d_33_ while applying the maximum loads of 200, 500, and 1000 μN. We found that the effective piezoelectric coefficients varied from 76 ± 2 pm/V to 96 ± 5 pm/V as shown in Fig. S5, from which, we conclude that the effective d_33_ of the core-shell micropillars is at least 1.85 times larger than that of the films. It should be noted that the converse piezoelectric coefficient is in principle the same as the direct piezoelectric coefficient^21^, and, therefore, we will use the term piezoelectric constant to account for both coefficients.

There are two potential reasons behind the enhancement of the piezoelectric constant of P(VDF-TrFE) core-shell micropillar. The first potential reason is that the shell of P(VDF-TrFE) core-shell micropillar generates a piezoelectric output from both the core and the side walls when a constant compressive load is applied to the structure. To unveil the contribution of each part of the pillar to the piezoelectric constant, we separately calculated the charge (Q) generations at the core and the shell of the pillar, respectively, based on the assumption that the contribution of each part adds linearly (Q_tot_ = Q_core_ + Q_shell_ = (d_33_^core^ + d_31_^shell^)F).
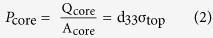

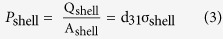
where P is the polarization charge per unit area, d is the piezoelectric constant, σ is the applied stress and A is the surface area. The subscripts, core and shell, refer to each part of the pillar. On the basis of Eq. (2) and (3), the generated charges from the shell are much larger than those from the core part in the P(VDF-TrFE) core-shell micropillar (see Eq. (S3)–(S6) for more detailed comparison). Therefore, we expect that the same effect applies to the effective converse piezoelectric coefficient of the micropillar where the converse d_33_ of the core and the converse d_31_ of the side wall add together to yield higher effective converse d_33_ of the whole structure.The second potential reason is the geometrical strain confinement effect in P(VDF-TrFE) core-shell structures. The micropillar geometry can be compared against thin film of P(VDF-TrFE) to determine which structure can accommodate larger strain for a given applied compressive load.

**Figure 4 f4:**
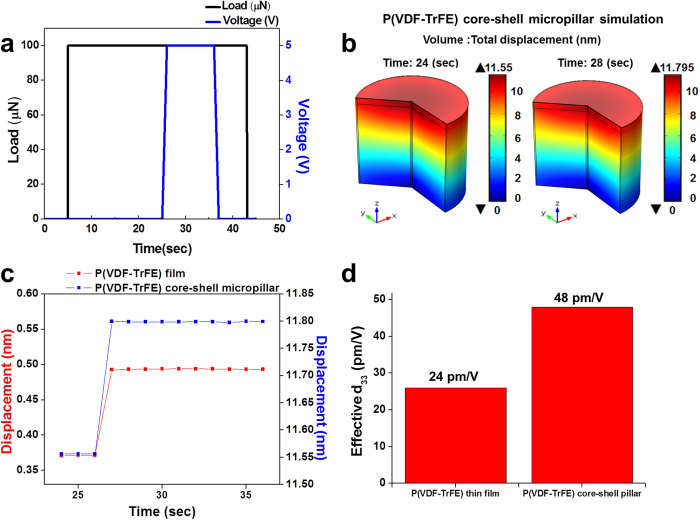
Numerical modeling of converse piezoelectric effects of P(VDF-TrFE) thin film and core-shell micropillar. (**a**) The trapezoid load function with loading-hold-unloading segments while applying an electrical bias voltage on the P(VDF-TrFE) thin film and the core-shell micropillar. (**b**) Change of calculated displacements in core-shell micropillar before and after applying bias voltage. (**c**) The calculated displacement changes of the P(VDF-TrFE) film (red squares) and the core-shell micropillar (blue squares) induced by an electric bias voltage of 5 V. (**d**) The effective converse piezoelectric constant d_33_ of the P(VDF-TrFE) film and the core-shell micropillar calculated using the model described in the text.

A numerical modeling was used to uncover the difference in the piezoelectric effect between the P(VDF-TrFE) film and core-shell micropillar when an external bias voltage is applied at the top surface as shown in Fig. 4. Here, the cross sectional area of the micropillar was chosen to be the area of the flat punch tip for thin film compression in order to keep the initial contact areas the same between the pillar and the thin film. The modeling was performed by using the finite element analysis software, COMSOL Multiphysics, with “Piezoelectric Devices (pzd)” module within the Structural Mechanics.

The parameter values for P(VDF-TrFE) were taken from the reported piezoelectric constants evaluated experimentally based on converse piezoelectric method^22^,^23^ while the properties for other materials including c-Si, Pt and PUA were taken from the literature^14^,^24^,^25^. A constant load was applied while the voltage was changed from 0 V to 5 V as shown in Fig. 4a. Upon application of voltage, the displacement field for the P(VDF-TrFE) core-shell micropillar changed as shown in Fig. 4b,[Fig f4]c where the net displacement change due to converse piezoelectric effect was 0.24 nm. The same loading conditions applied to the 1 μm thick P(VDF-TrFE) film on Si substrate resulted in a smaller net displacement change of 0.12 nm by the converse piezoelectric effect. Although the absolute piezoelectric constants are different from the experimentally measured ones, the enhancement of 2 times when comparing the pillar with the film is in accordance with experimental finding of 1.85 times (see Fig. S6 for more details). The discrepancy in the absolute values may come from the assumptions taken in the calculation such as elastic modulus and piezoelectric coefficients. However, the conclusion remains the same that the core-shell micropillar is expected to show larger converse piezoelectric effect due to the presence of the side walls and change in geometrical strain confinement effect. Other details of FEM modeling can be found in Supporting Information.

## Conclusion

In summary, we fabricated vertically aligned P(VDF-TrFE) core-shell micropillars that could overcome the structural limitation arising from the lack of stiffness in the P(VDF-TrFE) material. In addition, we were able to improve the piezoelectric effect by at least 1.85 times when compared to the thin film counterpart, which contributes to the more efficient current generation under a given stress, by making an effective use of the P(VDF-TrFE) thin top layer as well as the side walls. We attribute the enhancement of piezoelectric effects to the contributions from the shell component and the strain confinement effect. Accordingly, we believe that these flexible P(VDF-TrFE) core-shell micropillars can be used widely not only in nano- and micro-scale 3D pressure sensors (air or gas) but also in piezoelectric power generators.

## Methods

### Experimental details of fabrication of P(VDF-TrFE) core-shell structures

For the fabrication of the P(VDF-TrFE) core-shell micropillars, UV-curable polyurethane acrylate (PUA) elastomer (MINS series, Minuta Tech.), which has a relatively low viscosity (168 cPs at 25 °C) and a high modulus, was selected as the supporting material. The micro-patterned PUA pillars were fabricated via a UV imprinting process. A micro-patterned Si master mold was prepared and used to replicate the polyurethane acrylate (PUA) micropillar arrays with a 5 μm diameter, a 20 μm depth, and a 30 μm pitch using photolithography and a reactive ion etching (RIE) process. In order to prevent the master and the PUA elastomer from being stuck together, the surface of the Si master was treated with a liquid phase deposition of trichloro-(1*H*,1*H*,2*H*,2*H*-perfluorooctyl) silane (97%, Aldrich) for 1 h. Then, the UV-curable PUA elastomer was coated onto the Si master mold. The flexible and transparent 125 μm thick poly carbonate (PC) film with a high level of thermal stability up to 130 °C (Glastic® SCL, i-components Co., Ltd.) was surface-modified via plasma treatment in order to improve the adhesion at the PC film/PUA elastomer joint. Then, the PUA elastomer was covered with a back-supporting PC film. A slight rolling pressure was applied to the PC film to ensure a perfect infiltration of the elastomer into the trenches of the master template. The PC film was subsequently exposed to UV light (λ = 250 - 400 nm) for 150 s through the back-supporting PC film. After the UV curing, the PUA pillar structure was peeled from the master.

Then, the platinum bottom electrode was deposited on the PUA pillars via a sputtering process at room temperature at 2 mTorr and 20 W for 20 min under 50 sccm of Ar. For the uniform deposition of the Pt on the side walls of the PUA pillars, the PUA pillars were placed in a tilt holder at angle of 30 degrees with respect to the Pt target and were rotated by 90 degrees about the surface normal axis of the PUA pillars for four times.

For the piezoelectric layer deposition, we used the P(VDF-TrFE) copolymer powder (MSI, Inc.) consisting of 75 mol% VDF and 25 mol% TrFE 3 wt% and dissolved it in methyl ethyl ketone (MEK) using an ultrasonic treatment. The solution was spin-coated onto the Pt-coated PUA pillar arrays at a low rotation speed (600 rpm). In order to ensure conformal coating over the micron size pillars, we blocked the four edges of the sample using a 62.5 μm thick tape which is higher than the pillar height and rotated the sample at low speed (600 rpm). The solution was then evaporated during the post-annealing process at 125 °C for 2 h that led to a conformal pillar coating.

### Characterization of P(VDF-TrFE) core-shell structures

We confirmed the formation of vertically aligned Pt-coated PUA, and the P(VDF-TrFE) core-shell arrays using scanning electron microscopy (SEM). We also confirmed the formation of ferroelectric β-phase in the P(VDF-TrFE) layer via Fourier transform infrared (FTIR) spectroscopy in the attenuated total reflection (ATR) mode (IFS66V/S & HYPERION 3000, Bruker) and X-ray diffraction (XRD) (D/Max-2500with, RIGAKU Co.) with a 2θ scan at 40 KV and 80 mA. All thicknesses of the core-shell micropillar were determined via transmittance electron microscopy (TEM). Focused ion beam (FIB) milling was used to prepare a cross-sectional TEM specimen.

### Numerical simulation

The model was meshed using triangular elements, with smaller element size enough to keep the solution convergent. The simulation results were obtained using a time-dependent solver, and to control the error in each integration step, relative tolerance and absolute tolerance for the solution were chosen to be 10^−5^ and 10^−3^, respectively. Displacements at the most bottom surface of core-shell and thin film structure were constrained along the z-axis. All other functional boundary conditions in the simulations were comparable to experiments.

## Additional Information

**How to cite this article**: Choi, Y.-Y. *et al.* Vertically aligned P(VDF-TrFE) core-shell structureson flexible pillar arrays. *Sci. Rep.* **5**, 10728; doi: 10.1038/srep10728 (2015).

## Supplementary Material

Supplementary Information
