# Supplementary material for: Vertically aligned P(VDF-TrFE) core-shell structures on flexible pillar arrays
Source: Sci Rep. 2015 Jun 4;5:10728. doi: 10.1038/srep10728 (PMC4455118; doi:10.1038/srep10728)
Supplement: Supplementary Information [file srep10728-s1.pdf]

Supplementary Information

Vertically aligned P(VDF-TrFE) core-shell structures on flexible pillar arrays

Yoon-Young Choi[1, 2†], Tae-Gwang Yun[3†], Nadeem Qaiser[3], Haemin Paik[1], Hee Seok Roh[4], Jongin Hong[5], Seungbum Hong[1, 2*], Seung Min Han[3*], and Kwangsoo No[1*]

[1]Department of Materials Science and Engineering, Korea Advanced Institute of Science and Technology (KAIST), Daejeon 305-701, Korea

[2]Materials Science Division, Argonne National Laboratory, Lemont, IL 60439, USA

[3]Graduate School of Energy Environment Water Sustainability, Korea Advanced Institute of Science and Technology (KAIST), Daejeon 305-701, Korea

[4]Nuclear Engineering Division, Argonne National Laboratory, Lemont, IL 60439, USA

[5]Department of Chemistry, Chung-Ang University, Seoul, 156-756, Korea

† Equal contribution toward the article
* Co-corresponding author

* Corresponding authors: hong@anl.gov (S. Hong), smhan01@kaist.ac.kr (S. M. Han) and ksno@kaist.ac.kr (K. No).

1. Inspection of Pt and P(VDF-TrFE) layers deposited on PUA pillars

Fig. S1 presents the SEM images and EDS spectra of the Pt coated PUA micropillars and the P(VDF-TrFE) core-shell micropillars. Based on the SEM images, the pillar structures were maintained after each deposition step of the Pt and the P(VDF-TrFE) layer on the PUA pillars. The Pt peak, and the C and F peaks in the EDS spectra confirm the presence of the Pt and P(VDF-TrFE) layers on the pillar structures, respectively.

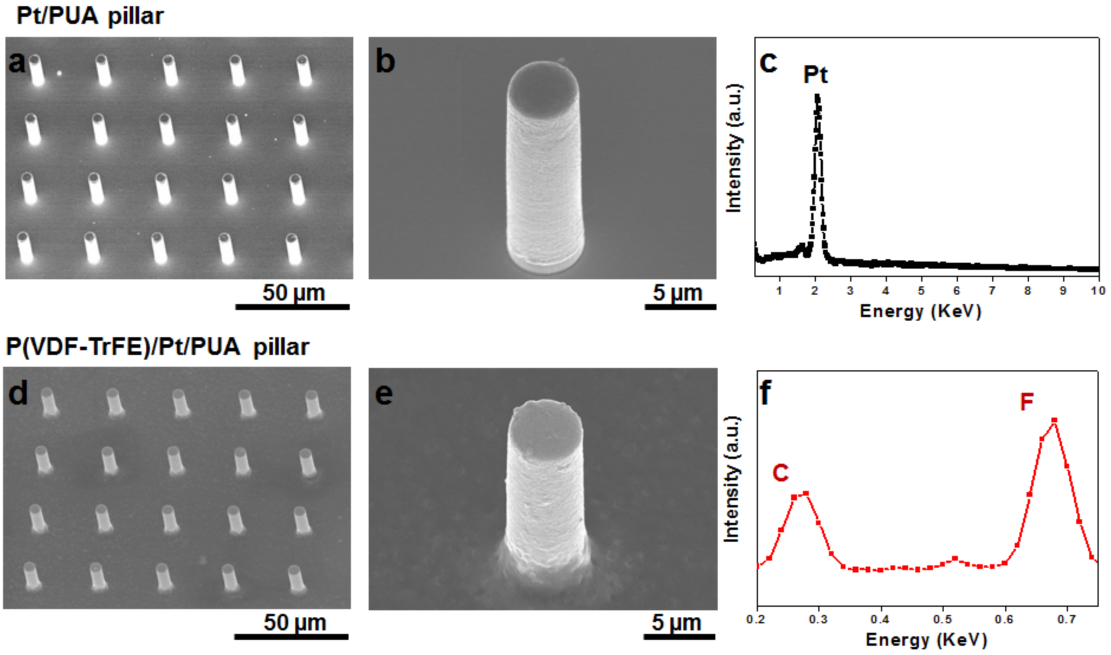

Figure S1. SEM images: (a) and (b) show the Pt coated PUA micropillars, and (d) and (e) show the P(VDF-TrFE) core-shell micropillars. EDS spectra: (c) and (f) are collected from a Pt coated PUA micropillar and a P(VDF-TrFE) core-shell micropillar, respectively.

2. Calculation of contact area based on unloading stiffness

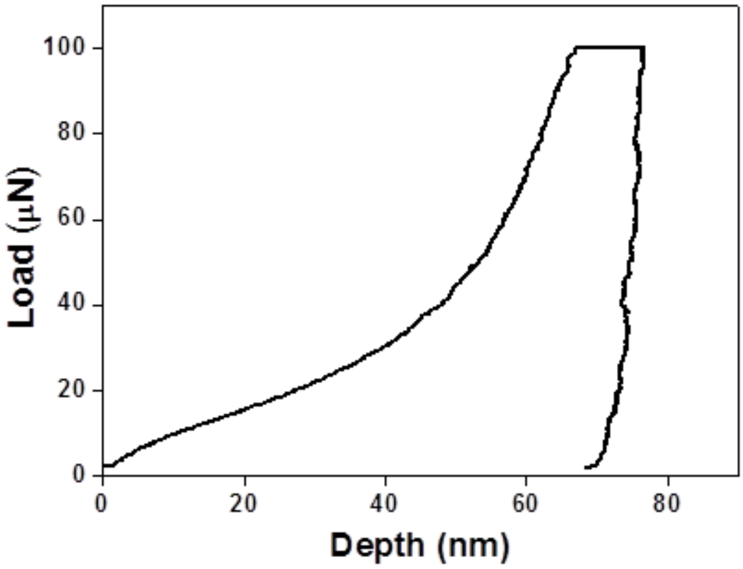

Figure S2. Force-displacement curve during multiple loading-unloading cycles.

In order to confirm that our data was collected with full contact to the micropillar, the contact area between the tip and the micropillar was evaluated from the unloading stiffnesses calculated from Fig. S2. The unloading stiffness is given by k=dP/dh, where P is the load, h is the displacement. According to Hooke's law,

$$\sigma = E_{avg}\varepsilon, \text{ or } \frac{P}{A_c} = E_{avg}\frac{h}{L_0}, \tag{S1}$$

where $L_0 = t_{P(VDF-TrFE)} + t_{pt} + t_{PUA}$, and $E_{avg} = \left[\frac{v_{P(VDF-TrFE)}}{E_{P(VDF-TrFE)}} + \frac{v_{pt}}{E_{pt}} + \frac{v_{PUA}}{E_{PUA}}\right]^{-1}$, and A_c is the cross-sectional area of the micropillar that is in contact with the tip,

Since $k = dP/dh = E_{avg}A_c/L_o$, A_c can be determined by the following equation:

$$A_c = \frac{kL_o}{E_{avg}} \tag{S2}$$

The calculated contact areas were similar to the cross-sectional area of the micropillar, which confirms that a full contact was successfully made.

3. Measurement of direct piezoelectric effect

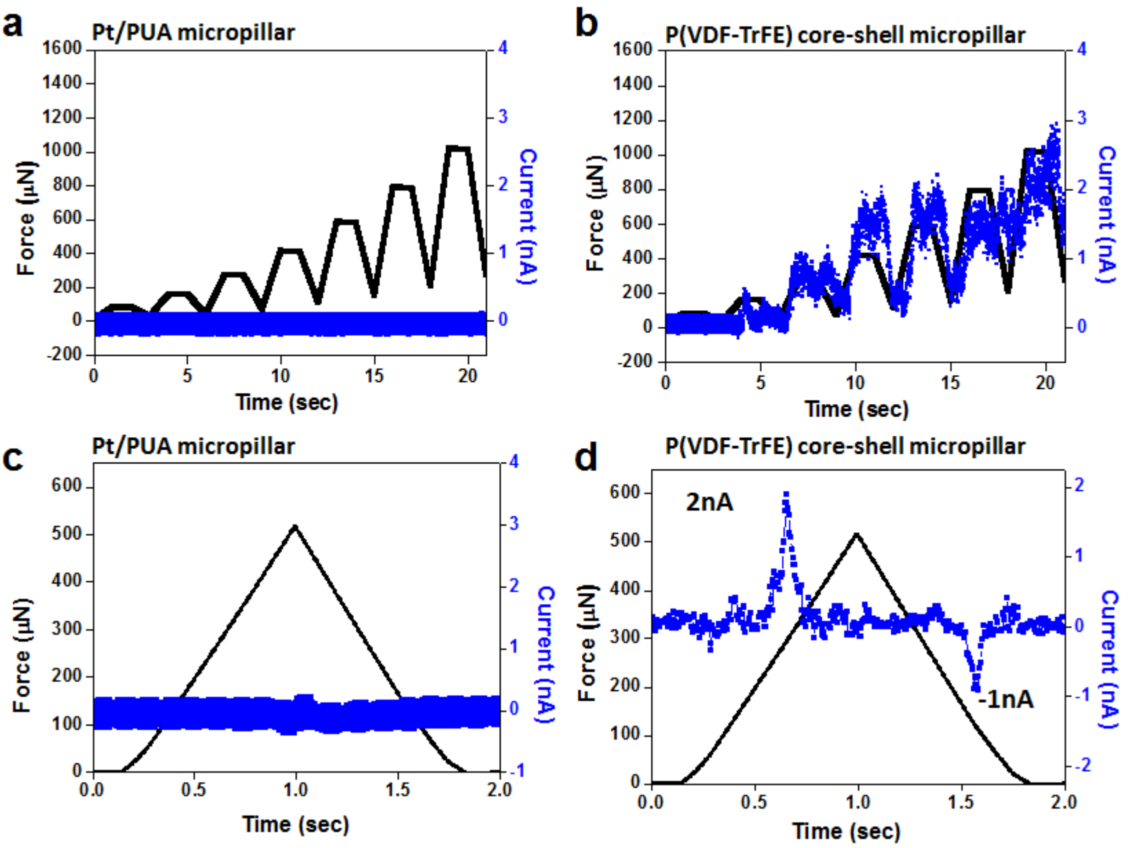

Figure S3. Characterization of current generations from an individual Pt/PUA and P(VDF-TrFE) core-shell micropillar. (a) Pt/PUA micropillar and (b) P(VDF-TrFE) core-shell micropillar under multiple loading-unloadings, (c) Pt/PUA and (d) P(VDF-TrFE) core-shell micropillar under triangular loading-unloading at a fast loading rate of 598.6 μN/s to determine impact current generations.

We attempted to measure the piezoelectric current generation of the P(VDF-TrFE) core-shell micropillar using a TI-750 triboindenter (Hysitron, Inc.) with a flat punch boron-doped diamond tip. The NanoECR instrument enabled simultaneous measurements of both the mechanical and the electrical properties. The current generations during the multiple loading-unloading nanoindentations were estimated, and the applied maximum force for each loading-unloading segment was gradually increased from 58 μN to 1016 μN as a function of time. When the applied force reached the maximum force of each step, the maximum force was maintained for 1 s followed

by subsequent unloading to 70 % of the applied maximum force for the segment (see Fig. S3a and S3b). In addition to collecting the piezoelectric current of the P(VDF-TrFE) core-shell micropillar, the same measurements were performed on the Pt/PUA without any P(VDF-TrFE) to verify that the generated current is indeed due to the piezoelectric generation from the P(VDF-TrFE) layer. As shown in Fig. S3a, there were no significant changes in the output current from the Pt/PUA structure. However, the generated maximum current for the P(VDF-TrFE) core-shell micropillar for each loading segment increased positively from 0 nA to a maximum of ~2.94 nA as the maximum force was increased from 58 μN to 1016 μN.

In Fig. S3c and S3d, a triangular loading-complete unloading was applied to the P(VDF-TrFE) core-shell micropillar in order to confirm the piezoelectric current generations under an impulse force, which is the type of the force loading typically found in the piezoelectric power generators. The maximum applied force was 515.3 μN at a fast loading-unloading rate of 598.6 μN/s in order to reduce the creep effect, which impedes the piezoelectric response of the material under slow mechanical stimuli. It was also confirmed that a positive current of ~2 nA was produced during the application of the compressive force while a negative current of ~1 nA was produced as the force was released.

However, we found that the current was generated even in holding segments when we applied multiple loading-unloadings to the P(VDF-TrFE) micropillar. After investigating the NanoECR instrument with the experts from Hysitron, we found that the measured piezoelectric current has a significant portion of an internal leakage current. Therefore, an alternative method, which is to measure the converse piezoelectric effect, is required to analyze the piezoelectric properties of P(VDF-TrFE) core-shell micropillars when using the NanoECR instrument.

4. **Measurement of displacement induced by applied bias under different loading conditions**

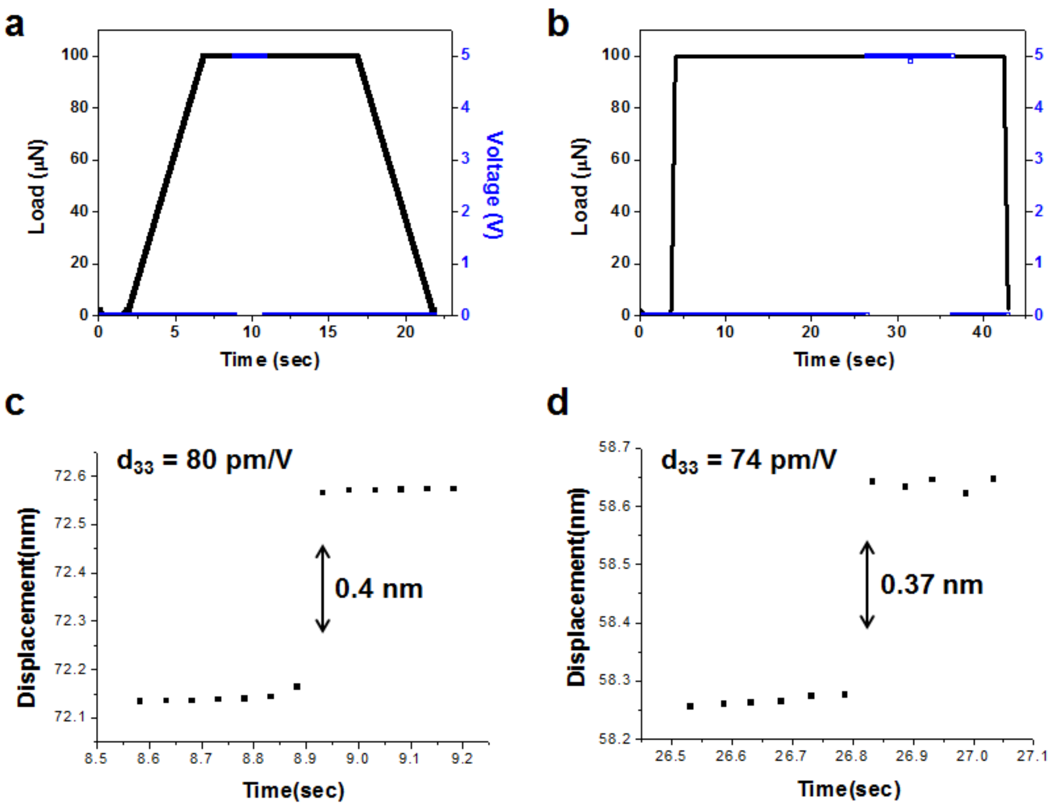

Figure S4. Plots of load and bias voltage as a function of time (a, b) and displacement of the tip in contact with the micropillar while applying bias of 5 V to the tip (c, d) under the loading conditions of (a) and (b).

Fig. S4a and S4b show the loading functions used to analyze both the piezoelectric displacement induced by the bias voltage and the polymer creep effect that can occur under a constant stress even without a bias voltage. Fig. S4c and S4d show the instantaneous displacement induced by a bias voltage of 5 V after a short and a long period of loading under grounded condition. The calculated d_{33} values were 80 and 74 pm/V, respectively, and an average value of ~75 pm/V was found after multiple tests on micropillars, indicative of minimal contribution of creep effect to the measured piezoelectric constants.

5. Measurement of piezoelectric constants under different loading conditions

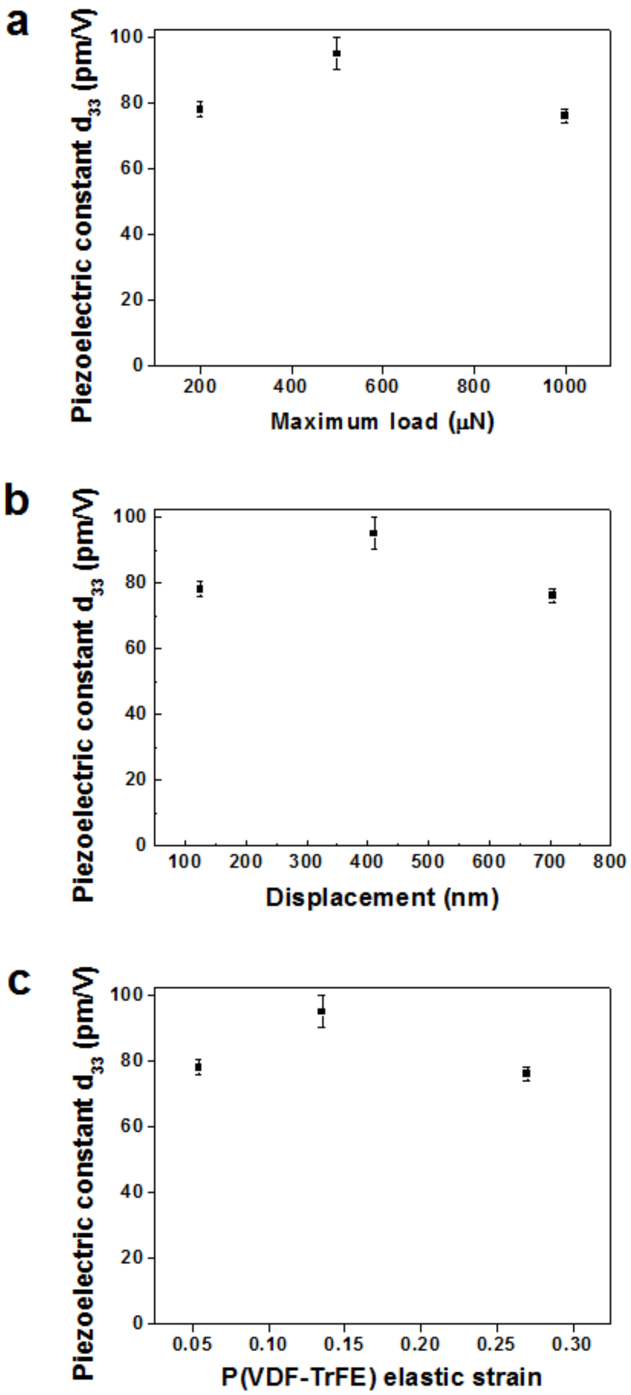

Figure S5. Plots of piezoelectric constant d_{33} value as a function of (a) maximum loading force, (b) displacement and (c) elastic strain.

6. Calculation of generated charges from the core and shell of the pillar

The charge generations at the core and the shell of the pillar were evaluated separately to determine the polarization contribution from the side walls. Here, we treat the structure as a core and a shell, where the shell consists of the P(VDF-TrFE) side wall and the core consists of composite of P(VDF-TrFE), Pt and PUA as explained below.

$$\sigma_T = v_{core}\sigma_{core} + v_{shell}\sigma_{shell} \tag{S3}$$

$$e = \frac{\sigma_T}{(v_{core}\,E_{core} + v_{shell}\,E_{shell})} \tag{S4}$$

$$\frac{1}{E_{core}} = \frac{\upsilon_{P(VDF-TrFE)}}{E_{P(VDF-TrFE)}} + \frac{\upsilon_{Pt}}{E_{Pt}} + \frac{\upsilon_{PUA}}{E_{PUA}} \tag{S5}$$

where σ_T is total stress of pillar, e is total strain of pillar, v_{core} and v_{shell} are volume fraction and E_{core} and E_{shell} are elastic modulus. E_{core} is the composite modulus for the P(VDF-TrFE)/Pt/PUA core structure that is calculated from an iso-stress condition of 2.1 GPa Eq. (S5). E_{shell} is simply the elastic modulus of P(VDF-TrFE) of 1.6 GPa. Once the total strain is determined, the stress at the core and the shell can then be determine at the maximum applied load 100 μN by

$$\sigma_{core} = E_{core}e_T \quad \sigma_{shell} = E_{shell}e_T \tag{S6}$$

The applied stress on the core and the shell are calculated to be 4.2 MPa, 3 MPa, respectively, from Eq. (S6). Since the core of pillar can be represented using an iso-stress model, $\sigma_{core} = \sigma_{core}^{P(VDF-TrFE)} = \sigma_{core}^{Pt} = \sigma_{core}^{PUA}$, and $\sigma_{core}^{P(VDF-TrFE)}$ is then given by 4.2 MPa.

Using the above values of stresses in the core and the shell P(VDF-TrFE) and using Eq. (2) and (3), the charge generation from the core and the shell are determined

to be 0.0032 pC and 0.0126 pC under 100 μN load, respectively. Therefore, this analysis confirmed that the charge generation from the shell is 4 times larger than the charge generation from the core.

7. Numerical modeling of converse piezoelectric effects of P(VDF-TrFE) thin film

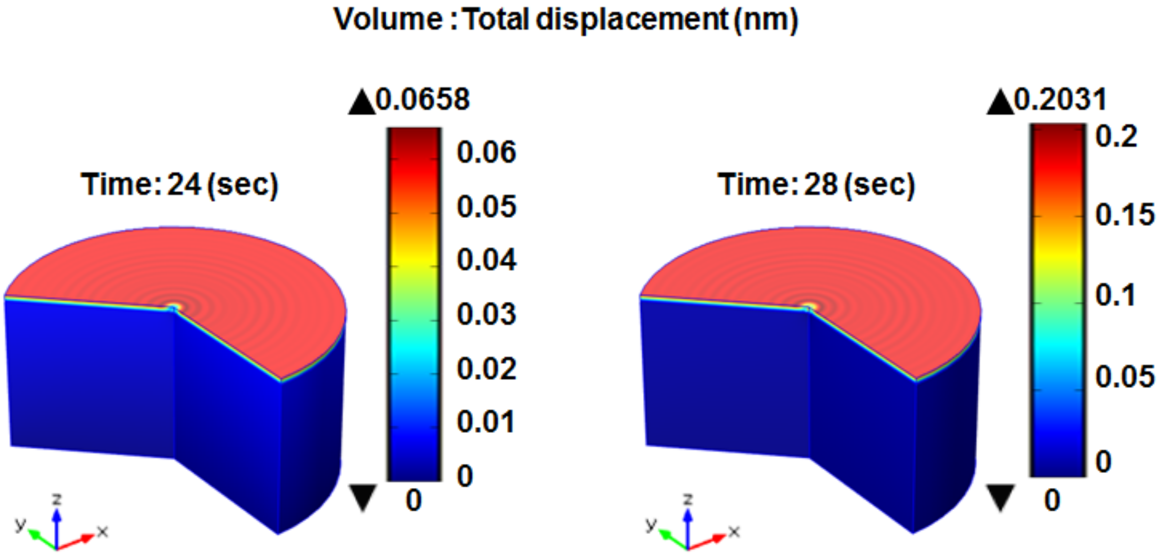

Figure S6. Change of calculated displacements in P(VDF-TrFE) thin film before and after applying bias voltage (5V) under trapezoid load function with loading-hold-unloading segments.

8. Visualization of self-polarization effect in the micropillar

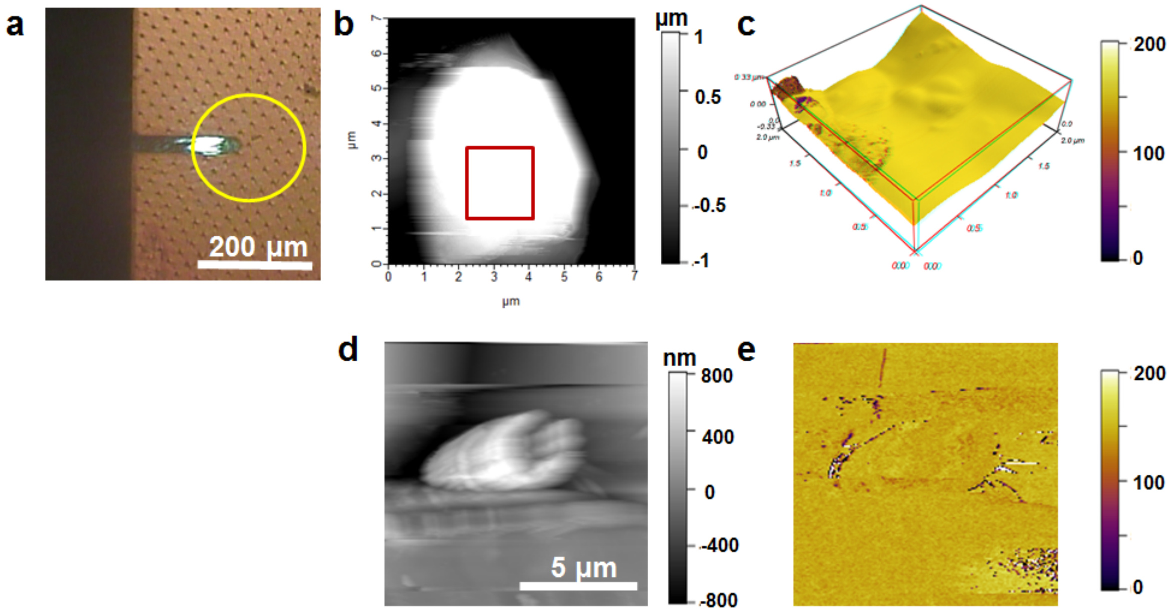

Figure S7. (a) Optical microscope image of P(VDF-TrFE) core-shell micropillars during PFM scanning, (b) topographic image of the top surface of the P(VDF-TrFE) core-shell micropillar, (c) three-dimensional representation of PFM phase overlaid on the topography of the P(VDF-TrFE) core-shell micropillar, (d) topographic image of the P(VDF-TrFE) core-shell micropillar, and (e) PFM phase image of the P(VDF-TrFE) core-shell micropillar.

In order to confirm the polarization direction of the micropillar, we conducted piezoresponse force microscopy (PFM) imaging on one of the pillars as shown in Fig. S7. After confirming the contact between the PFM tip and the core and the shell of the pillar as shown in Fig. S7a, S7b and S7d, we imaged both the topography and the PFM phase images of the surface of the P(VDF-TrFE) core-shell micropillar. In our PFM phase images, the dark region indicates the domains with upward polarization direction whilst the bright region indicates the domains with downward polarization direction. As such, we confirmed that most of the regions on the pillar was self-aligned from top to bottom electrode direction as expected from the piezoelectric current measurement. In addition, this result is consistent with previous results from Park *et al.*[1]

Reference

1. Park, M. *et al.* The piezoresponse force microscopy investigation of self-polarization alignment in poly(vinylidene fluoride-co-trifluoroethylene) ultrathin films. *Soft Matter* **8**, 1064-1069 (2012).
